# The Acceptability and Appropriateness of a Collaborative, Sport-Centered Mental Health Service Delivery Model for Competitive, and High-Performance Athletes

**DOI:** 10.3389/fspor.2021.686374

**Published:** 2021-11-08

**Authors:** Krista J. Van Slingerland, Natalie Durand-Bush

**Affiliations:** University of Ottawa, School of Human Kinetics, Ottawa, ON, Canada

**Keywords:** sports psychology, mental health care, acceptability, appropriateness, sport

## Abstract

The purpose of this study was to evaluate the acceptability and appropriateness of a sport-centered, collaborative mental health service delivery model implemented within the Canadian Center for Mental Health and Sport (CCMHS) over a period of 16 months. The study is situated within a larger Participatory Action Research (PAR) project to design, implement and evaluate the CCMHS. Primary data were collected from CCMHS practitioners (*n* = 10) and service-users (*n* = 6) through semi-structured interviews, as well as from CCMHS stakeholders (*n* = 13) during a project meeting, captured *via* meeting minutes. Secondary data derived from documents (e.g., clinical, policy, procedural; *n* = 48) created by the CCMHS team (i.e., practitioners, stakeholders, board of directors) during the Implementation Phase of the project were reviewed and analyzed to triangulate the primary data. The Framework Method was used to organize, integrate and interpret the dataset. Overall, results indicate that both practitioners and service-users found the model to be both acceptable and appropriate. In particular, practitioners' knowledge and experience working in sport, a robust intake process carried out by a centralized Care Coordinator, and the ease and flexibility afforded by virtual care delivery significantly contributed to positive perceptions of the model. Some challenges associated with interprofessional collaboration and mental health care costs were highlighted and perceived as potentially hindering the model's acceptability and appropriateness.

## Introduction

In September 2017, a group of stakeholders from the sport and mental health domains, including the two authors, commenced a Participatory Action Research (PAR) project to design, implement and evaluate a novel sport-focused mental health service delivery model applied within a national center that became the “Canadian Center for Mental Health and Sport” (CCMHS; Van Slingerland et al., 2019). Stakeholders critically examined the Canadian sport and mental healthcare landscapes to identify strengths and gaps, finding a dearth of opportunities for competitive and high-performance athletes to access acceptable, and appropriate mental health care informed by a sport lens (Van Slingerland et al., 2019). This finding was in line with an accruing body of evidence demonstrating the value and need to provide sport-informed mental health services and resources to address the unique needs and demands of the athletic population (Henriksen et al., 2019, 2020; Reardon et al., 2019). In response, the group designed a specialized collaborative, sport-centered mental health service delivery model (Van Slingerland et al., 2020a) and implemented it over a period of 16 months as part of a larger three-phase research project (i.e., Design Phase, Implementation Phase, Evaluation Phase; Van Slingerland et al., 2019). This study is linked to the Evaluation Phase of the project and its purpose was to evaluate the acceptability and appropriateness of the mental health service delivery model implemented within the CCMHS during the implementation phase based on service-user and practitioner perspectives, as well as CCMHS documentation.

## Acceptability and Appropriateness

Within the context of health care, the constructs of acceptability and appropriateness provide valuable insight into the quality of services provided. According to the World Health Organization (2021), quality health care is safe, effective, patient-centered, timely, efficient, and equitable, and results in more benefit than harm to patients. More specific to mental health, high quality mental health care services provide “accepted and relevant [syn. appropriate] clinical and non-clinical care aimed at reducing the impact of the disorder and improving the quality of life of people with mental disorders” (World Health Organization, 2003, p. 2). Thus, the quality of mental health service delivery is underpinned by notions of what is *acceptable* and *appropriate* care according to recipients and providers.

Acceptability is “a multifaceted construct that reflects the extent to which people delivering or receiving a healthcare intervention consider it to be appropriate, based on anticipated or experiential cognitive and emotional responses to the intervention” (Sekhon et al., 2017, p. 95). According to Sekhon et al. (2017), seven components inform service-user and provider assessments of acceptability: (a) affective attitude, (b) burden, (c) ethicality, (d) intervention coherence, (e) opportunity costs, (f) perceived effectiveness, and (g) self-efficacy. Definitions of each component are provided in Table 1.

**Table 1:** Components of acceptability within healthcare interventions (Sekhon et al., 2017).

**Component**	**Definition**
Affective attitude	How one feels about the care process
Burden	The perceived amount of effort required to participate in the care process
Ethicality	The extent to which care has a good fit with one's value system
Intervention coherence	The extent to which one understands the care process and how it is designed to work
Opportunity cost	The extent to which benefits, profits, or values must be given up to engage in the care process
Perceived effectiveness	The extent to which care is perceived to have achieved its purpose
Self-efficacy	The level of confidence one has to perform the behaviors required to participate in the care process

The acceptability of an intervention for service-users and providers is a key indicator of both the effectiveness and the success of implementation of healthcare services (Diepeveen et al., 2013). When service-users consider the care they receive to be acceptable, they are more likely to adhere to treatment protocols and benefit from improved clinical outcomes (Hommel et al., 2013). Regarding success, when practitioners deem a health care model or protocol to be acceptable, they are more likely to deliver it as it was designed (Proctor et al., 2009).

Appropriateness is another construct shedding light on the quality of healthcare interventions. According to the Canadian Medical Association (2015), appropriate care is “the right care, provided by the right providers, to the right patient, in the right place, at the right time, resulting in optimal quality care” (p. 2). Appropriateness has also been described as the perceived fit or relevance of a healthcare intervention in a particular context for a particular target audience (Peters et al., 2013). In the context of the current study, appropriateness was employed to reflect the fit or relevance of the collaborative, sport-centered mental health service delivery model implemented within the CCMHS for competitive and high-performance athletes.

The constructs of acceptability and appropriateness were put forward to the stakeholder group by the first author during the design phase of the larger PAR project mentioned above. The stakeholder group approved the use of these constructs to guide the evaluation phase of the research.

## The CCMHS Service Delivery Model

Health service delivery models or frameworks are sets of abstract concepts that, together, create a vision to guide health care practice (Alligood, 2002; Fawcett and Desanto-Madeya, 2013). Models vary across disciplines and according to clinical contexts. The CCMHS service delivery model was designed by 20 stakeholders through a collaborative process that translated stakeholders' thoughts and the relationships between these thoughts into an objective, visual representation using Group Concept Mapping (see Kane and Trochim, 2007; Van Slingerland et al., 2020a; GCM). The GCM exercise was informed by focus group discussions in which stakeholders critically examined the Canadian sport and mental health care systems to evaluate the availability and effectiveness of mental health care for competitive and high-performance athletes (Van Slingerland et al., 2019, 2020a). Stakeholders concluded that a number of factors (e.g., lack of practitioners with dual competencies in sport and mental health, stigma, perceived lack of trust and confidentiality, inadequate funding, unclear eligibility criteria, and intake/referral processes, geographical constraints) contributed to low help-seeking and access to care among Canadian athletes.

The GCM exercise resulted in the generation of 106 unique statements describing what elements ought to be included in a sport-specific mental health service delivery model and team operating in the Canadian context. Statements were organized into a six-cluster solution (i.e., Service Delivery, Business, Policy and Operations, Communications and Promotion, Education and Training, Partnerships, and Research) that provided a framework to develop the service delivery model and CCMHS (Van Slingerland et al., 2020a). The *Service Delivery* cluster included 41 statements that informed stakeholders' conceptualization of the CCMHS service delivery model (e.g., “practitioners in the CCMHS should have dual competencies in clinical psychology *and* sport,” “establish standardized eligibility criteria to access services within CCMHS and a referral plan for those who don't meet the criteria”). Additionally, the *Business, Policy, and Operations* cluster (*n* = 20 statements), outlined foundational infrastructures (e.g., legal, administrative, technological) required to establish and operate the CCMHS and included 11 statements that directly influenced the development of the model (e.g., “use an electronic health records system,” “retain clinic manager and other human resources as necessary”). The remaining clusters provided guidance to further develop the Center itself, and to support service provision.

Following the GCM exercise, the stakeholders formed working groups based on their expertise to further delineate the service delivery model, addressing statements associated with defining service-user eligibility criteria, identifying an electronic health records (EHR) system, establishing a physical location for the CCMHS, and developing a payment structure for service-users. The project leads (i.e., two manuscript authors), in collaboration with the established board of directors, addressed the other statements that did not fall within the scope of the aforementioned working groups, such as incorporating the CCMHS as a not-for-profit organization, creating a website, outlining characteristics of the service delivery model, developing the intake and referral process, establishing a hiring process, and securing a team of mental health care practitioners (Van Slingerland et al., 2020a). At the completion of the implementation phase of the three-phase project, 81% (*n* = 86) of the 106 statements resulting from the GCM exercise were fulfilled, including 83% (*n* = 34) of the 41 statements in the *Service Delivery* cluster and 90% (*n* = 18) of the 20 statements in the *Business, Policy and Operations* cluster. The remaining statements (e.g., “create educational program and standards to train specialists to have competencies in both sport and mental health,” “create alumni program that engages recovered athletes in peer-to peer-mentoring”) will be addressed in the future.

Following are key characteristics of the CCMHS service delivery model emerging from the Design and Implementation Phases of the research project that are of particular relevance for the current study focused on evaluating the acceptability and appropriateness of the model (i.e., Evaluation Phase). Should readers be interested in better understanding the process of care provision (e.g., intake, referral, care provision, outcomes), they are invited to consult Van Slingerland et al. (2020b) and Durand-Bush and Van Slingerland (2021).

### Sport-Centered Care

The availability for Canadian athletes to receive care from mental health providers with expertise in sport remains limited (Van Slingerland et al., 2019, 2020a). This is a significant gap because evidence suggests that there are unique interactions between sport, mental health, and mental illness necessitating specialized expertise (Reardon and Factor, 2010; Reardon et al., 2019; Henriksen et al., 2020). For example, competitive and high-performance sport can uniquely compromise athletes' mental health (e.g., disturbed sleep due to travel schedules, overtraining and burnout; Meeusen et al., 2013; Drew et al., 2018) and trigger or exacerbate mental illness (e.g., due to concussion, cessation of sport due to injury, maltreatment, pressure to conform to body norms; Neal et al., 2013; Reardon et al., 2019). Moreover, correct diagnosis of mental illness can be compromised by sport (e.g., adaptive eating for an endurance athlete may present as an eating disorder to a clinician who does not have sport experience), and traditional treatment modalities (e.g., psychopharmacological interventions) may have adverse effects on performance (e.g., due to ataxia or weight gain), or be a banned substance under World Anti-Doping Association regulations (Reardon and Factor, 2010; Reardon et al., 2019).

Research has shown that athletes may greatly benefit from working with mental health practitioners who understand the competitive sport context (Gavrilova and Donohue, 2018; Moesch et al., 2018; Jewett et al., 2020; Van Slingerland et al., 2020b). For example, Jewett et al. (2020) found that high-performance athletes who perceived their mental health challenges to be inextricably linked to their sport experience (e.g., sport was a significant stressor, trauma was sustained in sport, symptoms impaired performance), also expressed the need for a mental health practitioner who understood the intricacies of sport. This mounting body of evidence was the impetus for developing a ‘sport-centered' service delivery model including practitioners with knowledge and experience working in sport. This knowledge and experience were deemed essential to tailor therapeutic approaches to meet sport-specific demands and concerns such as competitive pressure, year-round training, injuries, transitions, peak and recovery periods, diet restrictions, team culture, traveling schedule, and anti-doping regulations (Reardon et al., 2019; Van Slingerland et al., 2019). To this end, job postings to hire practitioners for the CCMHS care team were explicit in asking about applicants' knowledge and competencies in sport. For example, postings denoted that experience in sport (e.g., as an athlete, coach) or working with athletes or other high-performing populations (e.g., physicians, military, lawyers) was an asset, and applicants were invited to complete an appendix outlining the nature of their sport experience (e.g., work with individuals and teams, skills employed).

### Collaborative Care

In the current Canadian context, there are several types of professionals educated and trained to provide services in the areas of mental health, mental illness, and mental performance (Van Slingerland et al., 2019). As such, multiple professions were targeted in the CCMHS service delivery model to provide mental health care in competitive and high-performance sport contexts. At the time of the implementation phase, CCMHS practitioners included clinical and registered psychologists, counselors, psychotherapists, mental performance consultants, a family physician, and a psychiatrist (Van Slingerland et al., 2020a). Collectively, these team members complemented each other's scope of practice and had the competencies to diagnose, treat, and prevent mental illness, manage and improve mental health, and address sport performance-related concerns with individuals, teams, and families. The CCMHS Care Coordinator played a central role within the model by completing intake assessments, assigning clients to care teams, serving as a neutral touch-point for clients, assisting practitioners in applying CCMHS policies and procedures, and managing data to support ongoing research and evidence-based practice (Van Slingerland et al., 2020b).

A standard feature of CCMHS care included the assignment of a *lead* and a *support* practitioner to each client's care team (Van Slingerland et al., 2020b). The rationale for this practice was to offer varied approaches and areas of specialization to guide care planning and decision-making, ensure availability in the event of a crisis, accommodate for different time zones and provincial restrictions to care provision, distribute workload and emotional burden, and encourage peer-to-peer learning and professional development (Durand-Bush and Van Slingerland, 2021). This interprofessional approach necessitates collaboration on the part of CCMHS practitioners. Collaboration is central to integrated, patient-centered care delivered by multidisciplinary health teams who apply their complementary expertise, knowledge, and skills to positively impact care outcomes (Sicotte et al., 2002; Nancarrow et al., 2013). Collaborative approaches to service delivery are also commonly applied in sport settings in order to optimize athletes' physical health and mental and athletic performance (Reid et al., 2004). Interprofessional collaboration requires (a) shared values, ethics, consciousness, and vision, (b) clearly defined roles and responsibilities fostering interaction and interdependence, and (c) consistent and coordinated processes and communication to facilitate teamwork (Enderby, 2002; Interprofessional Education Collaborative, 2016).

The collaborative aspect of the CCMHS service delivery model was critical in overcoming the siloed decision-making that can be characteristic of health services offered within the sport and general healthcare systems (Tinetti et al., 2016; Ekstrand et al., 2019). CCMHS policies and procedures that were created and adapted based on ongoing feedback facilitated collaboration, communication, and shared decision-making amongst CCMHS practitioners. These pertained to eligibility criteria, consent to access services, referrals, intake assessments, a web-based EHR system, a virtual care platform, session and team consult notes, and regular team meetings, to give some examples. The amount of collaboration between the practitioners assigned to a care team ranged on a continuum from independent parallel practice to interdependent co-provision of care (Jones and Way, 2006), depending on factors such as symptom severity and complexity as well as practitioners' availability, personal characteristics, and geographic location.

### Nationwide Service Provision

Pan-Canadian service provision was another important feature of the CCMHS model. Athletes are located all over Canada and they often travel across the country and abroad for both competition and training purposes. They must also relocate at times to work with different coaches and teams. As such, identifying a network of practitioners able to consistently and reliably provide inclusive and equitable services across provinces and territories in Canada was a priority in the development of the model. This was also deemed important to overcome interjurisdictional restrictions to the practice of psychology. This wide “network” approach has been adopted by high-performance sport systems around the world to service national team athletes (e.g., Moesch et al., 2018; Australian Institute of Sport, 2021; English Institute of Sport, 2021).

### Virtual and In-Person Care

Given the increase in popularity and availability of virtual mental health care services (Palylyk-Colwell and Argáez, 2018; Van Slingerland et al., 2020b) as well as the sheer size of Canada, the CCMHS model encompassed both in-person and virtual care options, enabling Canadian athletes to obtain services in a cost-effective, timely, and convenient manner, particularly when traveling. To this end, a secure and legally compliant^1^ videoconferencing software was purchased, and training was provided to practitioners prior to the implementation phase to provide safe and confidential services. While this modality is an ideal solution to meet face-to-face with athletes who are unable to attend in person, it requires an acceptable internet connection, technological literacy, and a living space that provides privacy. It may not be suitable for clients with severe mental illness (Madigan et al., 2020; Van Slingerland et al., 2020b).

In sum, collaborative models of care have been applied for decades to integrate mental health supports into primary care settings (Eghaneyan et al., 2014). Likewise, collaborative practice is commonly applied in sport settings as a strategy to provide integrated support to optimize athletes' physical health and performance (Reid et al., 2004). Until the current research was undertaken, a collaborative model to address the mental health needs of competitive and high-performance athletes had yet to be empirically designed, implemented and evaluated. Furthermore, a model centered on sport to increase the appropriateness and acceptability of care (Gavrilova and Donohue, 2018; Van Slingerland et al., 2019; Jewett et al., 2020) did not exist in the literature. The CCMHS sport-centered, collaborative service delivery model guiding nationwide in-person and virtual mental health care represents a first-of-its kind in the world. Assessing the acceptability and appropriateness of this novel model is thus imperative and was the purpose of the current study.

## Methodology

### Participatory Action Research

This study, one of three in a larger multi-phase project, was guided by a PAR framework. PAR is an approach to inquiry that mixes elements of participatory research (Chevalier and Buckles, 2013) and action research (Costello, 2003) to collaboratively create and apply knowledge to affect positive change in a community (Borg et al., 2012). The group of stakeholders who collaboratively designed the mental health service delivery model and CCMHS (Van Slingerland et al., 2020a) participated in the entire 48-month project at varying levels (e.g., consultation, arrival at group consensus, joint decision and action; Chevalier and Buckles, 2013). Through a *Collective Agreement* signed by stakeholders, the group agreed upon and operated under shared principles of engagement (e.g., respect and open communication, consensus decision-making). Importantly, the stakeholder group included current and former competitive and high-performance athletes (*n* = 12), mental health care service providers (*n* = 6), and service-users (*n* = 10; i.e., people who identify themselves as present or past users of mental health services) whose diverse perspectives created rich and meaningful dialogue. While action researchers facilitate the production and application of knowledge from the position of an “outsider,” participatory researchers are seen as stakeholders and participants themselves with valuable experiences to contribute to the pursuit of collaborative knowledge generation and change to the status quo (Herr and Anderson, 2005). In line with the PAR approach, the two manuscript authors, both active participants in the sport and mental health domains as researchers, practitioners and/or service-users, were included as participants in this study (see Van Slingerland et al., 2020b for an in-depth description of the authors' ties to sport and mental health) along with the CCMHS practitioners, stakeholders, and Board of Directors described in the next section. Notably, the two authors were also part of the stakeholder group that guided the larger project; thus, their interpretation of the data was infused with important contextual and experiential understanding accumulated throughout the larger project.

The process of doing PAR is complex, multi-facetted and outside the scope of this paper to fully address. Readers wishing to learn more about the processes followed to undertake this particular project are invited to consult previous articles stemming from the project (e.g., Van Slingerland et al., 2019, 2020a).

### Data Collection and Analysis

**Figure 1:**
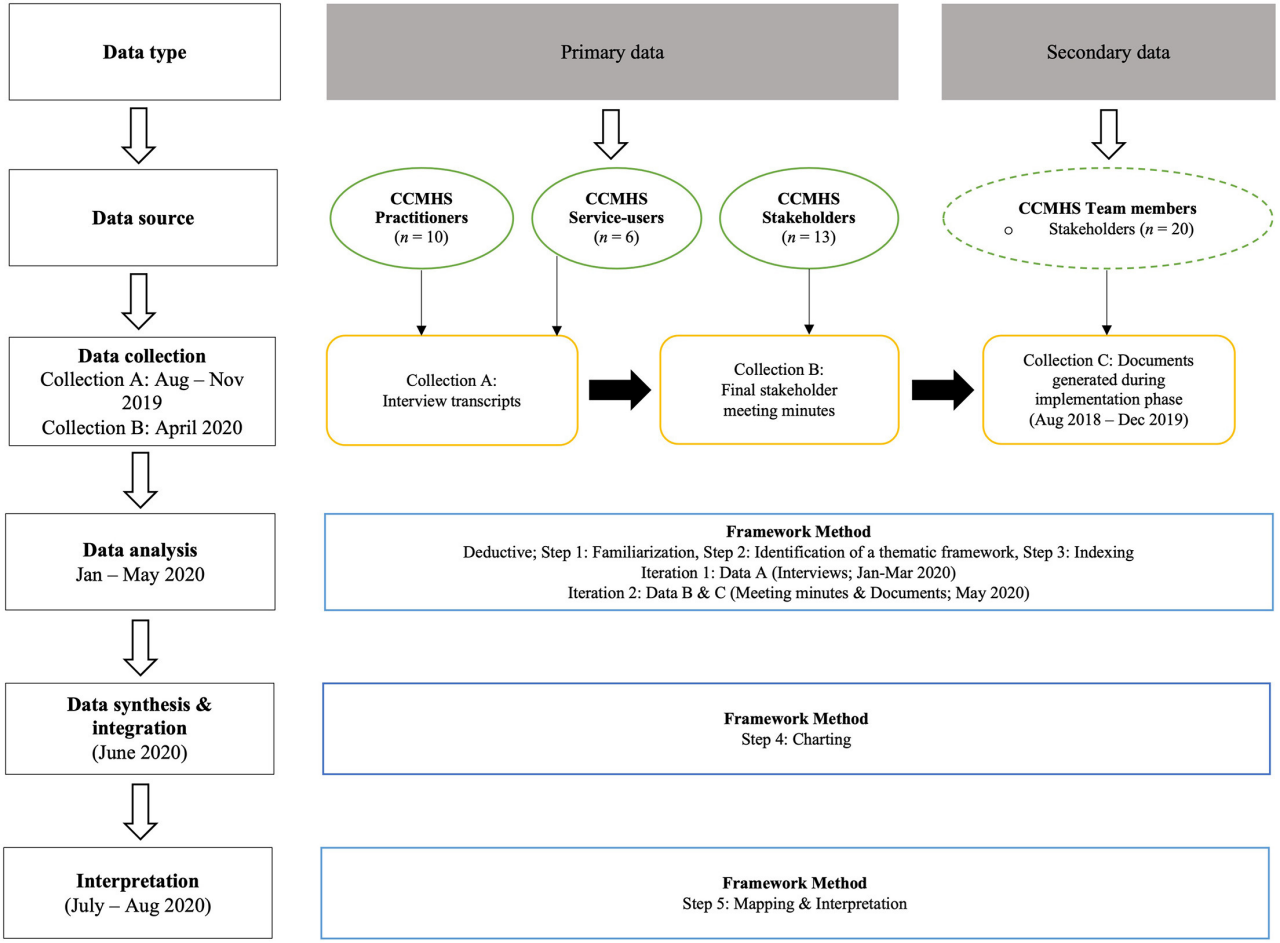
Overview of methods.

Ethical approval was obtained from the researchers' university Ethics Board to conduct this study. An overview of the data collection and analysis process is depicted in Figure 1. Both primary and secondary data were collected, using a three-step process (data collection A, B, C). Primary data were first collected sequentially from three sources: (1) CCMHS practitioners (see Van Slingerland et al., 2020b for a description of the full team), (2) CCMHS service-users (i.e., athletes), and (3) CCMHS stakeholders (see Van Slingerland et al., 2019 for details). Semi-structured interviews (data collection A, August—November 2019) served as the principal means to examine practitioner and service-user experiences and perceptions of the acceptability and appropriateness of the mental health service delivery model (Malson, 2010; Cheng and Clark, 2017). The results of a preliminary analysis of the interview data were then presented to CCMHS stakeholders during a meeting, held virtually due to the COVID-19 pandemic. Stakeholders' impressions and reflections were captured *via* meeting minutes (data collection B, April 2020).

Finally, to complement and triangulate interview data and stakeholder feedback, secondary data were also gathered (data collection C, May 2020) *via* documents (*N* = 86) produced by CCMHS team members (e.g., practitioners, stakeholders, board of directors) during the Implementation phase (August 2018—December 2019) of the larger project. The Framework Method (Ritchie and Spencer, 1994), an analytic approach that involves sorting and charting qualitative data into key themes and codes using a five-step process [(1) familiarization, (2) identification of a thematic framework, (3) indexing, (4) charting, (5) mapping and interpretation] was used to organize, integrate, and interpret the data.

Although the Framework Method provides a clear procedure, researchers are free to revisit steps to reconsider or rework ideas as the analytical process unfolds (Ritchie and Lewis, 2003). Likewise, PAR directs researchers and stakeholders to undertake a continuous and cyclical process of planning, action, observation, and reflection (Kemmis and McTaggart, 1988). An iterative approach was taken to move through the data collection and analysis processes in which data were collected, preliminarily analyzed, presented for collaborative reflection and feedback and then further analyzed. The pacing of this process is enumerated in Table 2.

**Table 2:** Pacing of data collection and analysis.

**Activity**	**Timing**	**Description**
Data collection A—Interviews	August—November 2019	Semi-structured interviews were conducted with practitioners and service-users.
Data analysis—Iteration 1	January—March 2020	Interview data were analyzed using the first three steps of the Framework Method, resulting in a thematic framework.
Data collection B—Stakeholder Meeting	April 2020	Results of *Data analysis—Iteration 1* were presented to stakeholders who reflected and provided commentary, captured in Meeting Minutes (Document 1).
Data collection C—Documents	May 2020	All documents included in the CCMHS electronic database and produced by CCMHS team members during the Implementation Phase (Aug 2018—Dec 2019) of the larger research project were identified (*N* = 86)
Data analysis –Iteration 2	May 2020	Documents identified in *Data collection C* were reviewed and excluded (*n* = 38) if they did not triangulate the data provided by participants interviewed for the study.^a^ Included documents (*n* = 48) were coded using the thematic framework (Steps 1 and 3 of Framework Method).
Data analysis—Synthesis and Integration	June 2020	Data were charted into a framework matrix in which each column represented a theme and each row represented a source of data (Step 4 of Framework Method).
Data analysis—Interpretation	July—August 2020	Data were examined using a “bigger picture” lens and interpreted based on convergence and divergence between sources and links across themes (Step 5 of Framework Method).

a *Any clinical documents included (i.e., session and team consult notes, intake summaries) from this point on pertained to the service-users who were interviewed for the study only*.

#### Data Collection A—Interviews

A total of 16 one-on-one semi-structured interviews (Brown and Lloyd, 2001) were conducted by the first author with CCMHS practitioners (*n* = 10) and service-users (*n* = 6) between August and November 2019. Interviews were conducted as participants were available, thus no particular order was followed. The sample of practitioners included registered/clinical psychologists (*n* = 3), certified counselors/psychotherapists (*n* = 4), and mental performance consultants (*n* = 3). Nine of the 10 practitioners were also professional members of the Canadian Sport Psychology Association. To be eligible to participate in the interviews, CCMHS practitioners had to have consented to do so, and were required to have delivered a minimum of three sessions of care to one or more service-users in order to have sufficient experience upon which to draw. Collectively, the practitioners had delivered 151 sessions of care to 45 athlete service-users at the time the interviews began.

In order for service-users to be eligible to participate in an interview and have adequate experiential data from which to draw, they had to have completed 3 or more care sessions with a CCMHS practitioner. Twenty-eight service-users met this threshold and were contacted to participate. However, only five female and one male athlete (*M*_age_ = 22.8 years) volunteered to be interviewed even though they had originally consented to be included in the study if they met criteria. This was not surprising given the busy schedule of athletes and the sensitivity of the topic being investigated (i.e., mental health care). Service-user participants competed at the provincial (*n* = 1), collegiate (*n* = 2), and international (*n* = 3) levels and sought CCMHS services to address symptoms associated with a variety of mental health disorders (e.g., depression, anxiety, ADHD, eating disorder). At the time of their interview, they had completed an average of 5 sessions with CCMHS practitioners. Five of them were still actively receiving care while one had completed the care program. A summary of service-user characteristics is presented in Table 3.

**Table 3:** Summary of service-user characteristics.

	**Service-User 1**	**Service-User 2**	**Service-User 3**	**Service-User 4**	**Service-User 5**	**Service-User 6**
Age	19	27	22	18	26	25
Gender	Female	Female	Male	Female	Female	Female
Sport type	Team	Team	Team	Team	Individual	Individual
Level	Collegiate	Provincial	International	Collegiate	International	International
Region	Atlantic Canada	Atlantic Canada	Central Canada	Central Canada	Western Canada	Western Canada
# of sessions	4+ intake	5+ intake	5+ intake	3+ intake	5+ intake	9+ intake

The interviews were informed by an interview guide, which was developed based on the components of Sekhon et al. (2017) framework of acceptability (see Table 1), and the elements of appropriate care enumerated in the Canadian Medical Association (2015) definition (i.e., (1) service characteristics [“right care”], (2) provider characteristics [“right provider”], (3) client characteristics [“right patient”], and (4) contextual characteristics [“right place and time”]). The first part of the interview was designed to elicit participants' perspectives on the seven components of acceptability in the context of the care they delivered or received through the CCMHS. As an example, practitioners were asked to describe any burden or opportunity cost they perceived to be associated with delivering care within the CCMHS model (e.g., “*As a member of the CCMHS care team, how much effort did you have to invest to provide adequate mental health care to athletes? To what extent did this team/context energize you and/or burden you?”)*. Service-users were asked a similar question (e.g., “*As an athlete receiving services at the CCMHS, how much effort did you have to invest to get adequate mental health care? To what extent did the team of practitioners/context energize you and/or burden you?”*).

The second part of the interview guide was designed to gather participants' perspectives on the extent to which the care delivered/received was appropriate. For example, practitioners were invited to address contextual characteristics [e.g., *What impact (if any) did the setting (physical location or e-platform) in which care was provided have on athlete outcomes (e.g., therapeutic alliance, adherence to the program, effectiveness of care?*]. Service-users responded to a similar question [e.g., “*What impact (if any) did the setting in which care was provided have on service delivery (e.g., therapeutic alliance, adherence to the program, effectiveness of care)?”*].

Although an interview guide was used, discussions remained flexible, allowing the first author to ask follow-up questions and participants to articulate their viewpoints in their own words, based on their experienced realities (Galletta, 2016). The interviews were conducted in-person (*n* = 3) and *via* a secure virtual platform (*n* = 13). They were audio-recorded and lasted 32 min on average.

#### Data Analysis—Iteration 1

A preliminary analysis of the interview data was undertaken in order to present findings to CCMHS stakeholders (*n* = 13) at a project meeting held virtually in April 2020. The data were examined using the first three steps of the Framework Method: (1) Familiarization, (2) Identification of a thematic framework, and (3) Indexing. Analysis began with a verbatim transcription of the interviews. Next, the first author familiarized herself with the interview transcripts, reading each one multiple times and re-listening to the audio recordings as necessary. The memo function in *NVivo* 12 was used to note initial thoughts and impressions, including any individual differences (e.g., geographic location) observed among participants that might influence their perspectives. Given the frameworks adopted to guide the study, a deductive approach to analysis was followed. The seven components of the acceptability framework (Sekhon et al., 2017) and four tenets of appropriateness (Canadian Medical Association, 2015) served as a thematic framework to organize the data. To index the data, the researcher used NVivo to code passages from the transcripts that spoke to one or more of the principal themes, while also allowing nuances within the broad themes to emerge (Gale et al., 2013). For example, *positive affect* and *negative affect* were codes relating to the broader category of *Affective Attitude* (Sekhon et al., 2017), which captured participants' feelings and emotions evoked by delivering or receiving care within the CCMHS model. Likewise, differences in the affective experiences of practitioners compared to service-users were noted.

#### Data Collection B—Stakeholder Meeting

To account for stakeholders' perspectives in the evaluation of the model, the broad themes, supported by quotes from participants, were presented to a subset (*n* = 13) of the original twenty-member stakeholder group (Van Slingerland et al., 2019), who met to share final reflections and close out the larger PAR project. Seven of the original stakeholders were unable to attend the meeting (*n* = 4) or were no longer engaged in the project (*n* = 3). Changes in the level of participation, including attrition, among stakeholders is common in PAR research as the conditions necessary for participation (e.g., time, trust amongst group members, professional obligations) fluctuate (Chevalier and Buckles, 2013). Stakeholder unavailability and attrition were unsurprising given the length of the larger project (32 months) within which this study was situated. The feedback provided by stakeholders during the meeting was captured within detailed minutes taken by the first author and confirmed by listening back to an audio recording of the proceedings, which lasted 150 min. The analysis of this data is described below (Data analysis—Iteration 2).

#### Data Collection C—Documents

A significant number of physical and electronic documents were produced by members of the CCMHS during the implementation phase of the larger PAR project. These documents (e.g., policy and procedural documents, electronic communications) provided valuable insight into processes and interactions between different groups involved within the CCMHS, successes, and challenges encountered (e.g., meeting minutes), and the outcomes of care (e.g., clinical documents) as the novel service delivery model was implemented. During *Data collection C*, documents that met the following eligibility criteria were identified and gathered for further analysis (Data analysis—Iteration 2): (a) they were created by a CCMHS team member (i.e., practitioners, stakeholders, members of the board of directors), (b) they were contained within the CCMHS' electronic database, (c) they were created during the implementation phase of the project (August 2018—December 2019). Eighty-six documents met these criteria. In addition to practitioners and stakeholders, members of the CCMHS Board of Directors (*n* = 7) contributed to document creation (e.g., policies/procedures). This group met quarterly to oversee the Centre's activities, develop organizational strategy, and ensure the organization complied with applicable legislation. While documents analyzed within the Framework Method are not typically written by researchers conducting an investigation (Bowen, 2009), many of the texts analyzed in the current study were written or influenced by the authors given that these individuals served as stakeholders commiserate with the PAR approach. These documents, along with the other documents produced by the CCMHS team, are labeled accordingly in Table 4.

**Table 4:** Documents analyzed.

**Document number**	**Name**	***n***	**Type**	**Author(s)**	**Year written**	**Document Purpose**	**Coding units/meaning assigned**
1	Meeting notes –Stakeholder Meeting #5	1	Meeting	N/A—Audio and visual recording of Zoom meeting	2020	Capture the content of a meeting with stakeholders where results of participant interviews were presented and analyzed	Burden
2-8	Meeting minutes—CCMHS Practitioner meetings	7	Meeting	N/A—Audio and visual recording of Zoom meeting	2018-2019	To capture the discussions that occurred within meetings	Affective Attitude; Intervention coherence; Perceived effectiveness
9	CCMHS Policies and Procedures	1	Policy/ procedural	CCMHS Board of Directors;CCMHS Practitioners	2018	Articulate the processes and procedures to be undertaken by CCMHS practitioners when delivering care	Burden; Intervention coherence
10	Authorization Form to Release Confidential Information	1	Policy/ procedural	Care Coordinator	2019	Allow clients to give consent to CCMHS practitioners to share information with other members of their circle of care (e.g., team physician)	Intervention coherence
							
11-42	Session and Team Consult Notes of the service-users interviewed	31	Clinical	CCMHS Practitioners	2018-2019	Summarize care sessions and consultations with CCMHS team members	Intervention coherence
43-48	Intake summaries of the service-users interviewed	6	Clinical	Care Coordinator	2018-2019	Summarize clients' presenting concerns, including scores on mental illness screening tools completed at intake	Intervention coherence; Perceived effectiveness

#### Data Analysis—Iteration 2

In the next phase of the data analysis, steps 1 and 3 of the Framework Method were applied to the documents gathered, which included the minutes produced from the stakeholder meeting (i.e., Data collection B and C). The first author first familiarized herself with the documents (step 1), determining if they met the following criterion to be further analyzed in this phase of the analysis: they triangulated the data provided by the practitioners and service-users who were interviewed for the study (i.e., confirmed or expanded the findings; Carter et al., 2014). Any clinical documents included (i.e., session and team consult notes, intake summaries) from this point on pertained to the service-users that were interviewed for the study only. Step 2 of the Framework Method (identification of a thematic framework) was unnecessary to repeat in this second iteration given the deductive approach used in the first iteration and the aim to triangulate the data rather than to produce new codes. In the end, 48 documents (55%) were included in the final analysis (Table 4). Excerpts from these documents were coded (step 3) using *NVivo* in light of the existing thematic framework. In addition to triangulation, the integration of data derived from CCMHS documents served to honor the PAR approach by including the ideas, actions, and voices of the CCMHS practitioners, stakeholders, and members of the board of directors who created them.

#### Data Analysis—Synthesis and Integration

In step 4 of the Framework Method, the coded passages (from interviews) and excerpts (from documents) were charted into a framework matrix in which each column represented a theme, and each row represented a source of data (e.g., practitioners, service-users, stakeholders, etc.). Organizing data in this way assisted the first author in reducing the data by clearly summarizing it categorically and identifying quotes and excerpts that were most illustrative of the theme (Gale et al., 2013).

#### Data Analysis—Interpretation

Once the matrix was populated, the first author was able to observe the “bigger picture” in step 5 of the Framework Method to identify convergence and divergence in the data, compare and contrast the responses of distinct groups, and corroborate interview findings with data gleaned from the documents. In this way, the entirety of the dataset was used to fulfill the purpose of the study. The first author shared her interpretation of the data with the second author and five other research colleagues who offered critical feedback and encouraged reflexivity (Smith and McGannon, 2018).

## Results

Results are organized according to the seven conceptual components of acceptability (Sekhon et al., 2017) and four conceptual components of appropriateness (Canadian Medical Association, 2015). The data gathered from the semi-structured interviews with practitioners and service-users provide the bulk of the evidence, and excerpts from or reference to CCMHS documents serve to triangulate these data.

### Acceptability

Results indicate that all facets of acceptability were satisfied by the CCMHS model. Practitioners and service-users gave examples of positive affect (e.g., trust), high self-efficacy (e.g., assisted by the care coordinator), and low burden (e.g., afforded to service-users through virtual care delivery). Furthermore, the care delivered was regarded as ethical (e.g., confidential), effective (e.g., due to sport focus), and coherent (e.g., service-users understood and applied the skills they learned in therapy). On the other hand, the model's acceptability was challenged by a certain level of negative affect (e.g., apprehension), burden (e.g., communication required between practitioners), and intervention coherence (e.g., collaboration among practitioners).

#### Affective Attitude

Affective attitude reflects how practitioners and service-users felt about the CCMHS care process. Participants reported experiencing a range of positive and negative feelings (e.g., feelings of trust, support, pride, uncertainty, apprehension, frustration) as a result of delivering or receiving care within the CCMHS service delivery model. For example, trust was addressed by both practitioners and service-users. Practitioner 2 shared: “I think [my experience] would have been different had I not known anybody [on the team]. I don't know if I would have felt as comfortable reaching out.” The team-based model decreased feelings of isolation and enhanced feelings of connectedness, comfort, and confidence in providing quality care: “It was helpful to feel part of a bigger system that we're all working toward the same goal and all working within the same population…that collaborative piece for me made it feel less isolating as a practitioner” (Practitioner 7).

Likewise, service-users discussed feelings of trust related to service provision. For example, Service-User 5 mentioned, “I trusted her because she has a sport background herself and has worked with other athletes. I felt that she just gets it.” Conversely, two service-users described feelings of trepidation since the CCMHS was a relatively unknown entity in the Canadian sport ecosystem: “[Seeking help] was like jumping off a cliff…I think that's always intimidating, but also because [the CCMHS] is so new and I had only really heard of the organization” (Service-User 6). Despite having initial apprehension to seek services, three of the six service-users described the CCMHS Care Coordinator as contributing to their level of trust and comfort:

“[The Care Coordinator] was so awesome! I was nervous. I had no idea what to expect with the intake interview. She was so friendly, and I felt like she was really approachable…In the past, it had been just myself and the mental performance coach and there wasn't really an unbiased middleman to help if I needed it. So, right off the bat I was like, okay this is legit!” (Service-User 5)

#### Burden

Burden refers to practitioners and service-users' perceptions of the amount of effort required to participate in the care process. The implementation of the new CCMHS service delivery model placed more burden on practitioners (e.g., upload session notes to the EHR system; CCMHS Care Policies and Procedures, Document 9) than on the service-users. Burden for practitioners was mainly related to respecting policies and procedures for communication (e.g., through the EHR system and virtual platform) and collaboration. For instance, Practitioner 1 indicated: “[The CCMHS] asked for practitioners to communicate when a client has exited care and I haven't been…it's not part of my process. I don't even think about it until we talk about it in a meeting” (Team Meeting 3, Document 4).

Similarly, virtual care provision challenged practitioners to develop novel skills, as discussed by Practitioner 7: “[Establishing a therapeutic alliance across a digital platform] was a challenge, but it was one that I had embraced, and I found it to be authentic.” Interactions *via* a screen required effort to capture service-users' full attention: “I'm hearing phones; they're stopping in the middle [of the session] because their texts are coming through. It's like “Okay, this is our therapy time, are you on do not disturb mode?” (Practitioner 3).

Three practitioners perceived the collaborative aspect of the model to create burden at times, as indicated by Practitioner 5: “Should I [collaborate] even though I don't need to? We don't want to overload people who have very heavy practices … when we chat it has to be for a reason.” Practitioner 1 also shared: “It's on us to create those links and use each other in that way to build relationships. I do think that's one of the weaknesses [of the model] versus if we were all in the same building.” Despite these challenges, practitioners demonstrated flexibility, patience and resilience throughout the implementation phase and nine out of ten perceived the value of working with the CCMHS team to outweigh the burden they experienced.

Service-users perceived very little burden associated with the care process. They described that engaging in therapy required work, however, the effort they invested was worthwhile because of the benefits they derived: “The level of care [has been] awesome. Sometimes you think it's going to be work, and it *is* work, but I enjoy doing it” (Service-User 6). Virtual care delivery was perceived by four of the six service-users as reducing the effort required to participate in care, as indicated by Service-User 3: “A lot of it's done virtually and that has its issues, but it also gives room for tons of flexibility, like being able to do things from the comfort of your own home.”

#### Ethicality

Ethicality refers to the extent to which care was perceived to have a good fit with practitioners and service-users' value system. None of the practitioners raised any ethical concerns; rather, they described elements of the model that heightened ethicality. For example, three practitioners discussed the care team assignment process as enhancing ethicality compared to other models of care provision:

There's really a lot of consideration that goes into the process. [The Care Coordinator] took the time to get to know this client … and thinks that this client can be a really great match with my approach and my values. I mean, you can't really get anything better than that. (Practitioner 1)

Similarly, the care team assignment process ensured that ethicality and duty of care with respect to client safety were met, as indicated by Practitioner 6: “I felt that we needed to continue [care] and [the client] needed more than a few sessions…but I would [need] a colleague physically located there so having a supporting practitioner locally helped remedy that [ethical dilemma] for me.” Ethical questions (e.g., “Is it appropriate to use virtual care for complex cases?,” Team Meeting 5, Document 6) were discussed with the practitioner team at meetings throughout the Implementation Phase.

All service-users indicated that CCMHS practitioners were able to facilitate psychologically safe, secure, and person-centered care that aligned with their values. For example, Service-User 3 shared: “There was never really any cause for concern with information that was being exchanged.” As a neutral entity operating independently from Canadian sport governing bodies, the confidentiality and safety of CCMHS services were highlighted, as explained by Service-User 5:

A lot of [health care providers in high-performance sport] have a hand in making decisions that could affect our career, like finding spots on the team or traveling. So, I don't want to go to these people and show them that I'm struggling and that I'm not strong enough to be on the team.

Four service-users discussed the significance of having a practitioner who understood and shared sport as a fundamental value:

was definitely more helpful than past providers…they were more realistic in terms of managing the issues that were going on with staying in sport. Because every time I've had an issue, I've had providers be like ‘Oh why don't you just take a step back?' (Service-User 2).

#### Intervention Coherence

Intervention coherence reflects the extent to which participants understood the care process and how it was designed to work. Three practitioners discussed initially feeling uncertain about implementing the novel model, however, this changed as they became more familiar with policies and procedures. For example, Practitioner 6 reported: “I think it's easier now that I feel more confident with the technology we're using. I'll be honest, it was stressful for me at the start.” Practitioners were reminded of procedures and given additional clarity about how to follow them in practice in each of the team meetings (e.g., “Remember to fill out the team consult notes form in the EHR after you have meetings/calls.”; Team Meeting 7, Document 8). Furthermore, the team was given the opportunity to provide ongoing feedback and suggest adjustments as new challenges arose (e.g., Authorization to Release Confidential Information form created to work with third party practitioners, Document 10). Overall, all 10 practitioners took steps to learn, understand, and contribute to refining CCMHS policies and procedures over time to optimize care. For example, Practitioner 7 explained how she learned to adapt to digital care provision: “Just using little gestures, I make sure I'm using eye contact, waves at the beginning [I try] to project warmth across the platform.”

Service-users' understanding of the care model, particularly the collaborative aspect, was less than that of practitioners, as Service-User 3 explained: “I'm not exactly sure how my [care] team was structured.” Even though the Care Coordinator explained the care model during each intake (e.g., Intake Summary 3, Document 45), service-users' lack of knowledge was not surprising given the variability in collaboration across practitioner teams and the focus on client needs during care. This did not appear to impact care outcomes, as captured in the following session note: “The client continues to note improved awareness of internal states” (Document 23).

#### Opportunity Cost

Opportunity cost reflects what practitioners and service-users had to give up (e.g., benefits, profits, values) in order to engage in the care process. Time and money were the two most prevalent elements given up by participants in order to deliver/receive care through the CCMHS. For example, the collaborative component of care, which was unremunerated, was an opportunity cost identified by some practitioners: “One of the challenges is the time and the money that it costs to have that collaborative conversation…With running your own business and having a seven-year-old and trying to stay healthy yourself…those 20 mins count!” (Practitioner 9).

However, nine of the 10 practitioners emphasized that the benefits exceeded the costs of being involved in the collaborative care team, as summarized by Practitioner 5: “I don't think there's a cost to it, I think it's an advantage! I think that the opportunities to collaborate, to share knowledge, to work together, and remove the barriers, are important.” The one practitioner, however, who did not perceive the return to be commiserate with the investment she made shared: “I put a lot of front-end time to train and attend meetings and get up to speed on everything. For the number of clients in return, I wouldn't say it was quite equal in terms of the effort out” (Practitioner 9).

Three service-users identified fees-for-service as an opportunity cost: “Just thinking about paying for services… You want to be better so you're investing all of this money… but the extra 200 dollars is actually a lot for athletes…especially, non-carded athletes” (Service-User 6). Data from the stakeholder meeting supported this, showing that 7% of referred service-users dropped out before care commenced, citing financial difficulties (Stakeholder Meeting 5, Document 1). This aligns with the findings of other researchers whose studies revealed that low socio-economic status is significantly related to psychotherapy dropout rates (Wierzbicki and Gene, 1993). Furthermore, ~2.3 million Canadians reported having unmet or partially met mental health care needs during the most recent census, most frequently citing not knowing where to access support, being too busy, or being unable to afford care as the reason they did not get help (Statistics Canada, 2018).

#### Perceived Effectiveness

Perceived effectiveness is the extent to which care was perceived to have achieved its purpose. Participants reported a high level of effectiveness regarding the service-delivery model. For example, Practitioner 7 shared: “I just had an athlete text me that they were able to meet their goal of increasing their mental performance and got accepted to the National Team!.” All practitioners reported being able to deliver effective services, three of them highlighting the collaborative component: “When my first client was someone who required more than just my support, a psychiatrist was brought in. And that certainly was a strength of the model” (Practitioner 2). Practitioner 3 explained the increased accessibility of care: “A plus of the Center is that [clients] do circumvent a long… probably 12 to 16 month wait list.” Four practitioners underscored that their sport background enhanced effectiveness: “I think [sport-specific knowledge] was critical. When we started to explore what options there were for ADHD, it was much more inspiring for him to know that [the team member] had the sport background as well.” (Practitioner 9).

Nonetheless, some challenges were noted including time zone management (e.g., “It has been difficult to schedule a meeting with one particular client because of the time zone difference and because that client is a high school student,” Team Meeting 4, Document 5) and interjurisdictional barriers to practice (e.g., “Discussed the idea of collaborative care between members residing in different provinces to work with limitations.,” Team Meeting 3, Document 4).

Service-users provided several examples of successes they experienced as a result of receiving care. For instance, Service-User 6 discussed learning to manage symptoms of anxiety: “So [we've been] working on how to get into the right mindset and if I'm really nervous, how I bring that back… It's helped a lot up front in terms of feeling more confident. Service-User 3 shared: “The biggest changes I've incorporated is working on managing stress levels, lowering anxiety levels and finding a balance.”

#### Self-Efficacy

Self-efficacy is the level of confidence practitioners and service-users had to perform the behaviors required to participate in the care process. Overall, self-efficacy was high amongst participants. From the practitioners' perspective, self-efficacy increased over time as they became more familiar with the collaborative care process. The physical distance between team members sometimes challenged their efficacy to work together: “[If] I knew people better or they knew me, I think it would probably make the collaborative piece work even better” (Practitioner 1). Technological difficulties also sometimes affected confidence, as reported by Practitioner 9: “You need to break up the session [when technological difficulties occur] …that's the only problem I think with distance.”

All six service-users consistently reported being able to apply the skills and tools they gained in therapy to both sport and life: “There's been tons of opportunities that I have been able to take [a skill] and put it into a workplace situation or a schooling situation” (Service-User 3). Nonetheless, one athlete shared how stigma still impedes the application of strategies learned in therapy: “I'm not really comfortable with my coach. I wouldn't be open enough to say ‘yeah I'm struggling with depression”' (Service-User 5).

### Appropriateness

Taken as a whole, the care provided or received through the CCMHS was perceived as appropriate [the right care (service characteristics), provided by the right practitioner (provider characteristics), to the right patient (client characteristics), in the right place, at the right time (contextual characteristics)].

#### Service Characteristics

For every service-user, the sport-specificity of care surfaced as a reason the CCMHS offered the “right care”: “The sport-focus was a big component for me. It definitely allowed it to be relatable… Now I can take those skills and apply them to real life” (Service-User 3). Service-users perceived practitioners' sport background as enhancing their understanding of athletes' environment and the expectations placed upon them. This, in turn, enhanced trust in the provider and skill transfer because practitioners were able to give relevant examples when imparting strategies and tools to enhance mental health and mental performance.

#### Provider Characteristics

Similarly, practitioners' knowledge, and understanding of what it means to be a competitive athlete, made them the “right provider”: “They eat, sleep, live that [sport] environment. And they don't have balance. So, a practitioner who doesn't understand that high-performance environment, I think would have unrealistic recommendations or expectations around balance” (Practitioner 7). The intake process, which allowed clients and practitioners to be “matched” based on a number of factors (e.g., client needs, symptom severity, location) also contributed to perceptions of being the “right provider” (Team Meeting 4, Document 5).

#### Client Characteristics

Clients' athletic identity, coupled with the recognition that mental health challenges were impacting sport performance made them the “right client” for the CCMHS: “What I'm doing with my sport is everything and—yeah, it's probably causing me some issues right now, but I would rather work through those issues than not be in sport” (Service-User 2). The “right client” was also associated with service-users who had the means to pay for care through private insurance or family support. This is the only factor that practitioners and service-users described as hindering the appropriateness of care: “I'm really sorry we lost that one [to financial difficulties] he so needed the Center… it breaks my heart because we want [to help] these people” (Practitioner 5).

#### Contextual Characteristics

**Four** service-users discussed why the “right place” for care to be delivered was virtually, in their own home: “I spend so much time training… I love that I can just sit at home and be eating or be stretching and chatting with [my practitioner] at the same time in the comfort of my own home” (Service-User 5). One service-user discussed the stigma attached to seeking mental health support in sport, noting how the social climate has changed recently, making it the “right time” for the CCMHS to offer its services: “I think with the Bell Let's Talk stuff and a lot of athletes coming out and being like, ‘It's okay” [to seek help]'. I was like, ‘Why not, we'll see what they say”' (Service-User 6).

## Discussion

The purpose of this study was to evaluate the acceptability and appropriateness of a sport-informed mental health care model implemented within the CCMHS. Overall, results demonstrate that care provided and received within the CCMHS service delivery model was acceptable and appropriate, and that each component of the model contributed uniquely to practitioner and service-user experiences. Some areas of improvement emerged, which have implications for further research and practice.

### Collaborative Care

Results indicated that the involvement of multiple professionals with complementary expertise, knowledge and skills in care provision was acceptable and appropriate to practitioners, and service-users. Specifically, the collaborative interdisciplinary approach contributed to the ethicality of the model, promoted the professional development of team members, and enabled Pan-Canadian service provision. Tools such as the EHR and clinical note templates as well as regular team meetings facilitated continuity of care amongst team members. According to research on interdisciplinary health teams, continuity of care is key to providing coherent and connected healthcare experiences for patients (e.g., Anderson and Helms, 1993; Busari et al., 2017). This is particularly important in sport as athletes frequently travel and can change teams during their career, potentially necessitating them to work with different health practitioners every time they relocate if there is no centralized or integrated service provision approach (e.g., Nikolić, 2020).

Research also shows that collaborative care provides organized opportunities for practitioners to learn from colleagues with diverse skillsets (e.g., *via* team meetings, grand rounds), leading to increased cooperation, communication, and comfort in implementing health interventions as a team (Feather et al., 2016; Horsley et al., 2016). Results of this study confirm this. Although there was a steep learning curve for practitioners at the beginning of the implementation phase, they shared that they valued the exchange of information, ongoing support, decreased sense of isolation, and unity in pursuit of high-quality patient care, made possible through the collaborative care model. The model provided a community of practice in which peer learning and support could occur. This has been shown to be beneficial in both healthcare (e.g., Markowski et al., 2021) and sport (e.g., Bertram et al., 2017) settings.

The collaborative component of the CCMHS model was also perceived to enhance the effectiveness and quality of care and ensure the “right provider” was accessible to service-users. A significant body of evidence has demonstrated that collaborative care models result in high-quality care and improved outcomes for patients with mental illness and substance use disorders (Mental Health Commission of Canada, (n.d.); Siobhan et al., 2013). A central role in the effectiveness and quality of care reported by participants was fulfilled by the CCMHS Care Coordinator. The Care Coordinator reportedly enhanced practitioners' understanding and ability to implement the model, promoted and ensured ethical service-provision, and increased service-users' trust in the quality, legitimacy and safety of services provided. This supports previous research showing that the care coordinator position is integral to mental health service provision within interdisciplinary settings and can positively impact patient recovery (Haggerty et al., 2003; Henriksen et al., 2020). Having a centralized Care Coordinator to manage care in a secure and confidential manner and serve as a neutral conduit between practitioners and service-users is novel in the provision of mental health services in sport in Canada. Readers who are interested in learning more about the robust intake-process implemented at the CCMHS are invited to consult the work of Van Slingerland et al. (2020b). Given the several benefits highlighted by participants, more research should specifically examine the Care Coordinator role so that this type of position can be leveraged in the future to facilitate the delivery of mental health care in sport.

Despite the aforementioned benefits, the collaborative component of the model was associated with some administrative burden as well as time and financial cost for some practitioners. The fact that practitioners were not remunerated for collaboration posed a challenge for some of them. This issue was highlighted by other researchers who noted that fee-for-service models disincentivize collaboration amongst practitioners by failing to remunerate interactions that do not directly involve patients (Wranik et al., 2017). An adequate funding model is required in the future so that practitioners can be compensated for their time spent engaging in collaborative care with both clients and the practitioner team. Another burden highlighted by some practitioners pertained to logistics or administrative tasks (e.g., learning how to use the EHR). Interestingly, administrative burden was found to be a significant source of stress for medical professionals and linked to burnout (National Academies of Sciences Engineering Medicine, 2019). Given the novelty of the current collaborative care model and the potential for mental health practitioners to experience burnout (Statistics Canada, 2021), the efficiency of CCMHS processes should be explored to minimize the administrative burden placed on practitioners without compromising ethical and professional obligations.

### Sport-Centered Care

Findings show that the specialized sport-centered nature of CCMHS services significantly contributed to perceptions of acceptability and appropriateness. This was perceived by participants to enhance affective attitude (e.g., trust, comfort), the ethicality of services (e.g., sport values aligned between practitioners and service-users), and the effectiveness of care. While research has shown that athletic identity can prevent athletes from seeking help for their mental health struggles (Gulliver et al., 2012), this study revealed that athletic identity may also contribute to help-seeking when sport-centered resources are available. Indeed, confidentiality and trust in mental health providers are known to facilitate help-seeking amongst young people (Gulliver et al., 2010). Consequently, integrating practitioners with knowledge and experience in sport, which is a unique feature of CCMHS's sport-centered mental health care model, may be a way to build the trust required amongst young athletes to seek help when in need.

According to a recent study with high-performance athletes, the sport knowledge of mental health care providers may be vital for not only help-seeking but also recovering from mental health challenges or disorders (Jewett et al., 2020). Given the salience of this component of care, further investigation is warranted to shed more light on the value and necessity of having a sport background when providing care to athletes and to determine if this varies across athletic populations and mental health disorders experienced. Furthermore, given the limited number of mental health practitioners specializing in sport in Canada (Van Slingerland et al., 2019), efforts should be made to provide adequate education and training to increase the network of available practitioners. This was a statement highlighted in the concept mapping activity that was performed to create the CCMHS (Van Slingerland et al., 2020a), and remains an outstanding endeavor.

### Nationwide Service Provision

The nature of nationwide service provision was perceived to have both benefits and drawbacks. While the pan-Canadian model facilitated the delivery of care to athletes across the country, it also contributed to practitioner burden and sometimes challenged their self-efficacy to collaborate at a distance. Previous research has highlighted the barriers that geographical distance poses to effective communication and collaboration amongst healthcare teams, underlining that proximity to coworkers impacts familiarity, ease of communication and cooperation (Cramton, 2001). One way to circumvent this is by increasing trust within collaborative teams. Indeed, trust in colleagues was found to be a key component of the successful implementation of collaborative care models (World Health Organization, 2016), and this was also highlighted by several practitioners in the current study. Further research on factors facilitating successful at-distance collaboration and trust without overly increasing practitioner burden is imperative, especially in light of the COVID-19 pandemic during which many health professionals are providing virtual care and experiencing exhaustion (Statistics Canada, 2021).

Although distance created challenges for practitioners, the dispersion of team members across the country was seen to enhance the ethicality of remote care provision to service-users experiencing more acute symptoms (e.g., self-harm or suicidal ideation). The collaborative and interdisciplinary aspects of the CCMHS model allowed lead practitioners to safely provide care from a distance while having a support practitioner on the care team who could provide in-person care if necessary. Some severe and complex mental health conditions are best addressed in person (Madigan et al., 2020; Van Slingerland et al., 2020b) and the deliberate care team structuring and coordination gave athletes living in both urban and rural communities the opportunity to quickly access their practitioner team based on their evolving needs. This type of ethical and convenient service delivery would likely not have been possible for athletes accessing care through the Canadian public health system given the excessively long wait times (Canadian Mental Health Association, 2017).

### Virtual and In-Person Care

As introduced in the previous section, results revealed that virtual care delivery was acceptable and appropriate to service-users who shared that receiving care *via* a secure online platform was effective and relieved some burden associated with participating in therapy. Likewise, other studies have revealed that virtual care can be effective in the treatment of mental illness (Langarizadeh et al., 2017; Palylyk-Colwell and Argáez, 2018; Van Slingerland et al., 2020b). While virtual care was appraised positively by service-users in the present study, it should be noted that these service-users were fortunate to have a safe and private space in their home in which to engage in therapy; this may not be the case for all athletes. Indeed, athletes could face privacy issues when traveling and sharing their room with others. Interestingly, a recent study demonstrated that athletes strategically use their smartphone to stay connected and effectively communicate with others (DesClouds and Durand-Bush, 2021). Consequently, the smartphone may be an effective tool for athletes to leverage to safely engage in virtual care, particularly when they are on the road.

Practitioners agreed that creating an authentic and successful therapeutic alliance over a digital platform was possible, however, they also noted that virtual care delivery created additional burden compared to face-to-face care, and that technological difficulties sometimes challenged their self-efficacy to deliver effective care. Given the exponential increase in online service provision as a result of the pandemic, researchers should more carefully examine the mechanisms and tools (e.g., smartphone) allowing mental health practitioners and service-users to successfully work together and achieve desired outcomes. Given that some service-users reported services to be cost prohibitive for them, attention should be focused on finding mechanisms to make care more affordable. Unfortunately, coverage (e.g., *via* private insurance and athlete assistance programs) for mental health care remains limited in Canada (Durand-Bush and Van Slingerland, 2021). Thus, lobbying the government as well as private donors and corporate sponsors to help subsidize care is essential. Langarizadeh et al. (2017) reported that “while being comparable to in-person services, telemental health care is particularly advantageous and inexpensive through the use of current technologies and adaptable designs, especially in isolated communities” (p. 240). It seems logical then to continue building on the current study findings to develop affordable virtual care options using the most effective and efficient available technologies.

## Strengths, Limitations, and Future Directions

The qualitative approach guiding the current study allowed for an in-depth investigation and understanding of the acceptability and appropriateness of the CCMHS service delivery model. It brought to light the experiences of practitioners and service-users and honored these experiences as true and legitimate evidence of the mental health service delivery process, as per the PAR approach. Furthermore, three types of triangulation means were employed [i.e., involvement of multiple researchers, data sources (practitioners, service-users, documents), and methods (analysis of interviews and documents through framework method); Carter et al., 2014] to ensure the reliability and trustworthiness of the findings.

Although efforts were made to recruit as many practitioners and service-users as possible, the sample was limited. It was difficult to recruit service-users to share their experiences, yet this was not surprising given that high-performance athletes have extremely busy schedules. Furthermore, stigma remains a barrier and it is common for athletes to want to keep their struggles private. Interestingly, this was a common reason that the service-users sought services *via* the CCMHS. As a third-party entity operating at arm's length of sport governing bodies with no political or financial influence, the confidentiality of service-users was a priority and was guaranteed. The need for confidentiality and the challenges inherent in discussing painful mental health-related experiences may help explain why service-users were reluctant to participate in the current study.

Since the larger PAR project began, the CCMHS model has been extended to include sport coaches and support staff, as well as performing artists (e.g., competitive dancers). Future studies should therefore include these populations as well. Given the novelty of the service delivery model and the expectation that it will evolve over time along with the team of practitioners, the model should be periodically evaluated using mixed methods and multiple sources of data. For example, a quantitative component could be introduced to track symptom remediation and other measurable therapeutic outcomes.

## Conclusion

The present study to evaluate the acceptability and appropriateness of a sport-informed collaborative mental health care model makes several significant contributions to research and practice. This model was the first of its kind to be systematically designed, implemented and evaluated to provide care to athletes experiencing mental health challenges and disorders. Overall, findings show that the model was acceptable and appropriate and features of the model (i.e., collaborative, sport-centered, nationwide, virtual and in-person care) should be maintained. Nonetheless, some aspects of the model can be improved, including remuneration for collaboration, subsidization of care for service-users, and efficiency of processes (e.g., use of the EHR, remote collaboration between practitioners who are not as familiar with the model and team).

Results of this study can be used to inform the provision of athlete mental health services in other competitive and high-performance contexts. For example, services provided at multisport events such as the Olympic or Paralympic Games can be set up to incorporate a collaborative mental health care team with expertise in sport, as well as both in-person and virtual care options. This is particularly salient for events in which a restrictive “bubble” is created to protect the health of athletes and staff as a result of the pandemic. Given that centralized coordination of care emerged as an important element of the model, allocating resources to hire a care coordinator to facilitate the management of information, staff, and mental health care is highly recommended, particularly within large sport systems and countries like Canada.

Evidence supporting the effectiveness of integrated mental health care models in sport is practically non-existent. This novel study significantly contributes to not only science but also the professional fields of sport and mental health. Results can be used as an incentive to invest funding and resources in (a) mental health services for sport participants, (b) education and training to ensure there is an adequate network of mental health practitioners with expertise in sport, and (c) research to examine the impact of specialized care on help-seeking, mental health, and performance outcomes.

## Data Availability Statement

The datasets presented in this article are not readily available because the data includes Personal Health Information and cannot be shared. Requests to access the datasets should be directed to Krista J. Van Slingerland, krista.vanslingerland@uottawa.ca.

## Ethics Statement

The studies involving human participants were reviewed and approved by University of Ottawa Research Ethics Board. The patients/participants provided their written informed consent to participate in this study. Written informed consent was obtained from the individual(s) for the publication of any potentially identifiable images or data included in this article.

## Author Contributions

KV conceived and carried out the research project in consultation with ND-B, and wrote the manuscript. ND-B supervised the project, provided critical feedback, and edited the manuscript. All authors contributed to the article and approved the submitted version.

## Conflict of Interest

The authors declare that the research was conducted in the absence of any commercial or financial relationships that could be construed as a potential conflict of interest.

## Publisher's Note

All claims expressed in this article are solely those of the authors and do not necessarily represent those of their affiliated organizations, or those of the publisher, the editors and the reviewers. Any product that may be evaluated in this article, or claim that may be made by its manufacturer, is not guaranteed or endorsed by the publisher.

## Footnotes

^1^Electronic health interventions in Canada must comply with regulations set out in the Personal Information Protection and Electronic Documents Act (a federal law relating to data privacy) and Health Information Protection Act (provincial legislation introduced to protect individuals' personal health information).
